# 

**Published:** 1877-05-20

**Authors:** 


ORIGINAL AND SELECTED ARTICLES—

Case of Cerebro Spinal Meningitis. By E. W. Lane, M. D., of Georgia ..... 111
Constipation. By L. B. Anderson, of Va. ..... 112
Gelsemium Sempervirens, Yellow Gels. (Wild Woodbine.) By I. J. M. Goss, A. M., M. D. ..... 113
The Skin. By L. P. Yandell, Jr., M. D. ..... 115
Version by the Vertex in Shoulder Presentations. By William H. Bulling, M. D. ..... 118
The Indications Afforded by the Pupil ..... 119
Treatment of Carbuncle. By J. H. Dibrell, Jr., M. D. ..... 120
Treatment of Puerperal Eclampsia ..... 121
Physiological Effects of Quinia ..... 122
Ophthalmolgy. Modified Operation for Cataract ..... 122

ABSTRACTS AND GLEANINGS—

The Method of Taxis, 123. Xanthium Strumarium, or Cocklebur, 124. Sub-acute Rheumatic Arthritis, 124. Typhoid Fever, How Produced, 125. Intra-Uterine Injections not Safe, 125. Extract of Logwood as a Disinfectant, 126. Sage's Catarrh Remedy, 126. Bright's Disease—New Diagnostic Symptom, 126. A Secret Remedy for Asthma, 126. Bromhydrate of Cicutine, 126. Treatment of Placenta Prævia, 127. Cold Baths in Infantile Diarrhœa, 127. Deep Injection of Chloroform in Sciatica, 127. Simple Means to Lessen the Pain of a Blister, 128. Piles—Immediate Cure by Igni-Puncture, 128. Treatment of Naso-pharyngeal Catarrh, 128. Hot Water for Treatment of Sick Stomach, 129. Spasmodic Asthma, 129. The Use of Chloroform, 129. Nitrous Oxid Gas, 130. Cow's Milk for Babies, 130. Tracheotomy with the Thermocautery, 131. Chromic Acid in the Treatment of Ulcerating Granulations of the Os Uteri, 131. Canderna on the Treatment of Acute Intestinal Catarrh in Infants and Sucklings, 131. Treatment of Vesical Hemmorrhage, 131. Rhus-Poisoning, 132. Electricity in Herpes Zoster, Cancer, etc., 132. Bacteria—Their Nature, 132. Bromhydrate of Conia, 132. An Electuary against Tapeworm, 132. Curability of Syphilis, 132.

PRACTICAL NOTES—

Quinine Pills, 133. Wine Bitters, 133. Spermatorrhœa, 133. Neurargic Pill, 133. Diabetes, 134. Chronic Sore Throat, 134. Follicular Pharyngitis, 134. Anti-emetic, 134.

SCIENTIFIC ITEMS—

Malleability of Gold, 134. The Planet Jupiter, 134. Evolution, 134.

EDITORIAL AND MISCELLANEOUS—

Special Notice, 135. The Medical Association of Georgia, 135. Hemorrhagic Malarial Fever, and our Journal, 136. Consultations, 136. Merrell, Thorp & Loyd, Cincinnati, 136. Opium Antidotes, 137. Vaseline in Nasal Discharges, 137. Provisional Association of America, 137. Osteoclast, 137. Dr. Barry's Address, 137. Blue Glass Cure, 137. The Georgia Pharmaceutical Association, 137. The State Medical Association of Alabama, 137. Arkansas Medical Association, 138. Medical Convention of Texas, 138.

BOOK NOTICES—

Modern Therapeutics, 138.

